# Tug of War games and PDEs on graphs with applications in image and high dimensional data processing

**DOI:** 10.1038/s41598-023-32354-5

**Published:** 2023-04-13

**Authors:** Hamza Ennaji, Yvain Quéau, Abderrahim Elmoataz

**Affiliations:** UNICAEN, ENSICAEN, CNRS, GREYC, Normandie Univ, 14000 Caen, France

**Keywords:** Applied mathematics, Computational science, Image processing

## Abstract

The aim of this note is to revisit the connections between some stochastic games, namely Tug-of-War games, and a class of nonlocal PDEs on graphs. We consider a general formulation of Tug-of-War games which is shown to be related to many classical PDEs in the continuous setting. We transcribe these equations on graphs using ad hoc differential operators and we show that it covers several nonlocal PDEs on graphs such as $\infty$-Laplacian, game *p*-Laplacian and the eikonal equation. This unifying mathematical framework allows us to easily design simple algorithms to solve several inverse problems in imaging and data science, with a particular focus on cultural heritage and medical imaging.

## Introduction

**Figure 1:**
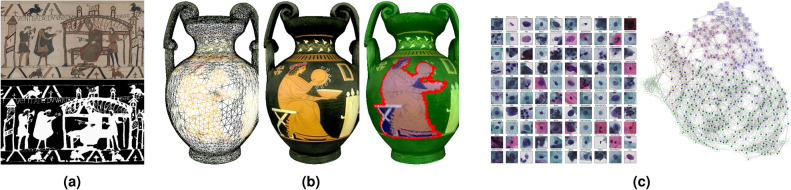
Examples of N-dimensional graphs, and of data processing problems which can be applied to them. (**a**) A 2D grid graph representing a color image, and the 2D segmentation of this image; (**b**) a 3D triangular graph representing a colored point cloud, and the 3D segmentation of this point cloud; (**c**) a ND point could graph constructed from a database of cells, and the data clustering of this graph. All these problems can be tackled using the same unified mathematical framework described in the present paper.

Local and nonlocal partial differential equations (PDEs) play a central role in the mathematical analysis and modelling of phenomena arising in physics, biology, economics, image and signal processing, computer vision, etc. Nonlocal PDEs compare favourably to the classical ones, thanks to their ability to preserve geometric and repetitive structures. Yet, the numerical approximation of classical PDEs, based on finite differences (FD), finite elements (FE) or finite volumes (FV) has a certain limitation especially when the computational domain is irregular or is a graph with arbitrary topology. Consequently, designing new methods for processing and analyzing data on graphs has been the object of many works. We mention among others^1–7^ where the authors propose the adaptation of many continuous PDEs and variational models such as the total variation flow, mean curvature flow and Hamilton-Jacobi equations to the framework of graphs. Naturally, this made discretizing and solving PDEs on graphs and networks gain attention and interest due to numerous applications in imaging, computer vision and machine learning, where data are given in the form of graphs or functions defined on graphs (see Fig. 1). Examples of such applications include, but are not limited to, segmentation, filtering, semi-supervised clustering, and classification.

In such settings, data points are vertices of the graph and are connected by edges if sufficiently close in a certain ground metric. Using discrete vector calculus^1,8,9^, one defines finite difference operators which are analogous to the differential operators in the continuous setting. This mimetic approach allows recovering many tools and results in the discrete level. This has been applied, e.g., for the $\infty$-Laplacian, the eikonal equation and the mean curvature flow^8,10,11^. Yet, each particular application requires resorting to a particular PDE, hence such approachs remain difficult to manipulate for non-experts.

On the other hand, the approximation of PDEs has recently taken advantage of the emergence of techniques based on deterministic or stochastic games such as the Tug-of-War (TOW) game related to the $\infty$-Laplacian^12,13^ and the *p*-Laplacian^14^. In particular, this formalism has been considered for studying the existence and regularity of a class of PDEs^15^. The main tool to make a link between the TOW games and PDEs is the dynamic programming principle (DPP): at each step of the game, the value function at some point can be obtained by summing up all the possible outcomes.

The main contribution of this paper is to propose general dynamic programming equations related to general TOW games, both in static and time-dependent case (c.f. Eqs. (5)–(6)). Using discrete calculus on graphs, we show that these equations are related to PDEs on Euclidean graphs, then we extend this to general weighted graphs c.f. (10). In particular, this allows us to extend the results of the preliminary work^16^ by covering both elliptic and parabolic PDEs as well as their game interpretation on general weighted graphs which we interpret as nonlocal TOW games. Moreover, we give the main arguments ensuring the existence and uniqueness of solutions to (10). Then, we will present in “Simple algorithms for inverse problems” section a few simple algorithms based on averaging operators which can be designed to solve inverse problems in image, 3D points clouds and data classification based on the transcriptions of such equations on graphs. In “Applications” section, we will provide numerical examples to illustrate the proposed method on real-life problems arising from cultural heritage and medical imaging, before drawing our conclusions in “Discussion” section.

## From TOW to PDEs on graphs

### TOW games and continuous PDEs

Let us briefly recall the notion of TOW as introduced by Peres and Sheffield^14^. The TOW game is a two player random turn zero-sum game played on a domain $\Omega \subset \mathbb {R}^N$ with a running payoff function $h:\Omega \rightarrow \mathbb {R}$ and a payoff function *g* defined on $\partial \Omega$. A token is placed at an initial position $x_0\in \Omega$ and each player can move the token to a new position in an $\epsilon$-neighborhood of the current position (typically, an open ball $B_{\epsilon }(x_k)$ if the token is at position $x_k$), for a fixed step size $\epsilon >0$. A fair coin is tossed, if player I wins, the token is moved to the position $x_{k}^{\textrm{I}}$, otherwise to the position $x_{k}^{\textrm{II}}$. The process continues until the token reaches some position $x_f\in \partial \Omega$. In that case, the game stops and player ${\textrm{I}}$’s payoff is $g(x_f) + \epsilon ^2 \mathop {\sum }\limits _{i=1}^{f-1} h(x_i)$. Since the game is zero-sum, player ${\textrm{I}}$ will try to maximize the payoff while player ${\textrm{II}}$ will try to minimize it. For this game, the DPP reduces to

1 $$\begin{aligned} \left\{ \begin{aligned} u_{\epsilon }(x)&= \frac{1}{2}\Big \{\sup _{y\in B_{\epsilon }(x)} u_{\epsilon }(y) + \inf _{y\in B_{\epsilon }(x)} u_{\epsilon }(y)\Big \}+ {{\epsilon ^2}} h(x)~&\text{ in }~\Omega ,\\ u_{\epsilon }(x)&= g(x)~&\text{ on }~\partial \Omega . \end{aligned} \right. \end{aligned}$$

Moreover, it is shown^12^ that the limit of $u_\epsilon$ as $\epsilon \rightarrow 0$ solves the normalized $\infty$-Poisson equation

2 $$\begin{aligned} \left\{ \begin{array}{ll} -\Delta _{\infty }^{N} u(x)= h(x)~&\quad\text{in}\quad\Omega ,\\ u(x)= g(x)&\quad\text{on}\quad\partial \Omega , \end{array} \right. \end{aligned}$$

where the normalized infinite Laplacian is given by $\Delta _{\infty }^{N} u = \vert \nabla u\vert ^{-2} \Delta _{\infty } u$, $\Delta _{\infty } u = \mathop {\sum }\limits _{i,j}\partial _{i}u \, \partial _{ij}u \, \partial _{j}u$.

There exist several variants of this game. A first example consists in playing the previous game with a certain probability $\beta \in [0,1]$, such that the token is moved to a position $x_{k}^{\textrm{I}}$ or $x_{k}^{\textrm{II}}$ with probability $\beta$, and to a random position in $B_{\epsilon }(x)$ with probability $1-\beta$. This version is called the TOW with noise^17,18^, and it is connected to the DPP3with 
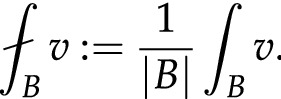
. Moreover, setting $\beta = \frac{p-2}{p+N}$, $p \ge 2$, and defining the normalized *p*-Laplacian according to $\Delta _{p}^{N} u = \frac{1}{p}\vert \nabla u\vert ^{2-p}{\text {div}}(\vert \nabla u\vert ^{p-2}\nabla u)$, the limit as $\epsilon \rightarrow 0$ of $u_\epsilon$ solves the following *p*-Laplace equation:

4 $$\begin{aligned} \left\{ \begin{array}{ll} -\Delta _{p}^{N} u(x)= h(x)&\quad\text{in}\quad\Omega ,\\ u(x)= g(x)&\quad\text{on}\quad\partial \Omega . \end{array} \right. \end{aligned}$$

This variant generalizes the classical TOW game, in the sense that (3) comes down to (1) when $\beta = 1$, and similarly, (4) comes down (at least formally) to (2), as $\mathop {\lim }\limits _{\beta \rightarrow 1} p = \mathop {\lim }\limits _{\beta \rightarrow 1} \frac{2 + \beta N}{1 - \beta } = \infty$.

**General TOW game:** Now, let us consider an even more general version as follows. Assume that each player chooses a position with probabilities $\alpha$ and $\beta$, respectively, and that the game position moves with a uniform probability $\gamma$, with $\frac{\alpha +\beta }{2} + \gamma = 1$. In this case, the DPP reads5This game can even be extended to the case where the probabilities $\alpha$, $\beta$ and $\gamma$ depend on both time and position $(t,x)\in [0,T]\times \Omega := \Omega _{T}$, where $T>0$. Therein, the value function of the game satisfies6In the particular case where $\alpha (x,t) = \beta (x,t)$, we obtain a space and time-dependent extension of (3). Moreover, setting $\alpha (x,t)=\beta (x,t) = \frac{p(x,t)-2}{p(x,t)+N}$ and $\gamma (x,t) = \frac{N+2}{p(x,t)+N}$, $p \ge 2$, the game is related to a normalized *p*(*x*, *t*)-Laplace equation^19^:

$$\begin{aligned} (N+p(x,t))\partial _t u(x,t) = \Delta _{p(x,t)}^{N} u(x,t) + h(x,t), \end{aligned}$$

which generalizes (4). However, the interpretation of (5) and (6) in terms of PDEs, in the most general case where $\alpha$, $\beta$ and $\gamma$ are only required to satisfy $\frac{\alpha +\beta }{2} + \gamma = 1$, is less straightforward. In the next paragraph, we prepare the main ingredients for this interpretation, which will also help us transcribing TOW games and the related PDEs to the framework of graphs.

### Reminders on differential operators on graphs

#### **Definition 1**

(Weighted graph) A weighted graph $\mathcal {G}= (\mathcal {V},\mathcal {E},w)$ is a collection of vertices $\mathcal {V}$, edges $\mathcal {E}\subset \mathcal {V}\times \mathcal {V}$ and edges weights $w:\mathcal {V}\times \mathcal {V}\rightarrow \mathbb {R}$ with $w(x,y)>0$ if $(x,y)\in \mathcal {E}$ and $w(x,y) = 0$ otherwise. We assume that $\mathcal {G}$ is finite (i.e., $\mathcal {V}$ consists of a finite number of points), simple (i.e., without loops and multiple edges), connected and undirected, that is $w(x,y) = w(y,x)$ if $(x,y)\in \mathcal {E}$. We write $x\sim y$ if $(x,y)\in \mathcal {E}$ and we denote by $I(x):=\{y\in \mathcal {V}: x\sim y\}$ the set of neighbors of *x*.

Given a function $u:\mathcal {V}\rightarrow \mathbb {R}$, we recall the definition of its gradients.

#### **Definition 2**

(*p*-eikonal operators) 

The discrete upwind nonlocal weighted gradients of *u* are defined by

$$\begin{aligned} \nabla _{w}^{\pm }u(x) = (\partial _{y}^{\pm }u(x))_{y\in \mathcal {V}}^{T}, \end{aligned}$$

where $$\partial _{y}^{\pm }u(x) = \left( \sqrt{w(x,y)}(u(y)-u(x))\right) ^{\pm },~ a^{+} = \max (a,0)$$ and $$a^{-} = \max (-a,0)$$.

Its $\mathcal {L}_p$ norm is defined as

$$\begin{aligned} \Vert \nabla _{w}^{\pm }u(x)\Vert _{p} ={\left\{ \begin{array}{ll} \max _{y\in I(x)} \left( \sqrt{w(x,y)}(u(y)-u(x))^{\pm }\right) ~\text{ for }~p= \infty \\ \Vert \bigl (\nabla _w^\pm u\bigr )(x)\Vert _p = \biggl [\sum \limits _{y \in I(x)} w(x,y)^{p/2}\big (u(y) - u(x)\bigr )^\pm \biggr ]^{\tfrac{1}{p}}~\text{ for }~1\le p<\infty . \end{array}\right. } \end{aligned}$$

#### **Definition 3**

(Laplacian operators on graphs)

1. The 2-Laplacian on graph is defined by
  $$\begin{aligned} (\Delta _{w,2}u)(x) = \frac{\sum _{y\in I(x)}w(x,y)u(y)}{\sum _{y\in I(x)} w(x,y)} - u(x). \end{aligned}$$
2. The $\infty$-Laplacian on graph is defined by
  $$\begin{aligned} (\Delta _{w,\infty }u)(x) = \frac{1}{2}\Big (\Vert \nabla _{w}^{+}u(x)\Vert _{\infty } - \Vert \nabla _{w}^{-}u(x)\Vert _{\infty }\Big ). \end{aligned}$$
3. For $2\le p<\infty$, the game *p*-Laplacian on graph is defined by
  $$\begin{aligned} (\Delta _{w,p}^{G}u)(x) = \frac{p-2}{p} \Delta _{w,\infty } u(x) + \frac{2}{p}\Delta _{w,2} u(x). \end{aligned}$$

### Connections between TOW games and PDEs on Euclidean graphs

We are now ready to introduce the transcription of the TOW games (5) and (6) in terms of PDEs on graphs. Let us consider an Euclidean graph $\mathcal {G}= (\mathcal {V},\mathcal {E},w)$ with $\mathcal {V}= \Omega \subset \mathbb {R}^N$ and a weight function

$$\begin{aligned} w(x,y) = {\left\{ \begin{array}{ll} 1~\text{ if }~\vert x-y\vert \le \epsilon ,\\ 0~\text{ otherwise }. \end{array}\right. } \end{aligned}$$

Then, using Definition 2, we easily get

7 $$\begin{aligned} \begin{aligned} \max _{y\in I(x)} u(y)&= \Vert \nabla _{w}^{+}u(x)\Vert _{\infty } + u(x),\\ \min _{y\in I(x)} u(y)&= u(x) - \Vert \nabla _{w}^{-}u(x)\Vert _{\infty }.\\ \end{aligned} \end{aligned}$$

Plugging (7) in (5), we obtain the following interpretation of the generalized TOW game (5) in terms of a PDE:

8 $$\begin{aligned} -\Delta _{\alpha ,\beta ,\gamma }u(x) =h(x), \end{aligned}$$

where $$\Delta _{\alpha ,\beta ,\gamma } u(x) = \frac{\alpha }{2} \Vert \nabla _{w}^{+}u(x)\Vert _{\infty } - \frac{\beta }{2} \Vert \nabla _{w}^{-}u(x)\Vert _{\infty }+\gamma \Delta _{w,2}u(x)$$.

Similarly, in the case of the time-dependent TOW game (6), we obtain the following parabolic PDE:

9 $$\begin{aligned} \partial _t u(x,t)=\Delta _{\alpha (x,t),\beta (x,t),\gamma (x,t)}u(x,t) +h(x,t). \end{aligned}$$

As can be seen in Table 1, by taking different values of $\alpha , \beta$ and $\gamma$ in the TOW games (5) and (6), we are able to recover different well-known PDEs on graphs. In order to apply this unifying mathematical framework to real-world imaging or data processing problems, it only remains to have at hand a practical way to discretize such PDEs, on graphs which are not necessarily Euclidean. This is discussed in the next paragraph.

### Extension to General Weighted Graphs

We have seen that on Euclidean graphs, PDEs of the form (8) are related to some classical TOW games. A natural question that one may ask is what can be said in the case of general weighted graphs, e.g., images or 3D point clouds, where the weights are not induced by a metric. To this end, let us consider a general weighted graph $\mathcal {G}= (\mathcal {V},\mathcal {E},w)$, and recall^20^ the following notations for nonlocal dilation, nonlocal erosion and nonlocal mean, respectively:

$$\begin{aligned} \begin{aligned} \text {NLD}(u)(x) = \Vert \nabla _{w}^{+}u(x)\Vert _{\infty } + u(x)&=\ u(x)+ \max _{y\in I(x)} \left( \sqrt{w(x,y)}(u(y)-u(x))^{+}\right) ,\\ \text {NLE}(u)(x) = u(x) - \Vert \nabla _{w}^{-}u(x)\Vert _{\infty }&=\ u(x)- \max _{y\in I(x)} \left( \sqrt{w(x,y)}(u(y)-u(x))^{-}\right) ,\\ \text {NLM}(u)(x) = u(x) + \Delta _{w,2} u(x)&=\ \frac{\sum _{y\in I(x)}w(x,y)u(y)}{\sum _{y\in I(x)} w(x,y)}, \end{aligned} \end{aligned}$$

which come down to the classical morphological dilation, erosion and mean when $w \equiv 1$. Indeed, in this case

$$\begin{aligned} \text {NLD}(u)(x) = \max _{y\in I(x)} u(y),~\text {NLE}(u)(x) = \min _{y\in I(x)} u(y),~\text{ and }~\text {NLM}(u)(x) = \frac{\sum _{y\in I(x)}u(y)}{card (I(x))}. \end{aligned}$$

Then, defining the following nonlocal averaging operator:

$$\begin{aligned} \text {NLA}(u):= \frac{\alpha }{2}\text {NLD}(u) + \frac{\beta }{2}\text {NLE}(u) + \gamma \text {NLM}(u), \end{aligned}$$

Equations (8) and (9) can be rewritten as

10 $$\begin{aligned} u(x) - \text {NLA}(u)(x) = h(x), ~~\text{ and }~\partial _t u = \text {NLA}(u)(x) -u(x) + h(x), \end{aligned}$$

This rewriting provides a practical way to discretize such nonlocal PDEs on general weighted graphs, as it suffices in practice to implement the nonlocal mathematical morphology operators above.

**Table 1:** A large class of PDEs on graphs recovered from (8)–(9), which are obtained by taking particular parameter values in the TOW games (5)–(6).

TOW game parameters	Elliptic PDE	Parabolic PDE
$\alpha = \gamma = 0$ and $\beta = 1$	Eikonal equation: $\Vert \nabla _{w}^{-}u(x,t)\Vert _{\infty } = h(x)$	$\partial _t u(x,t)=\Vert \nabla _{w}^{-}u(x)\Vert _{\infty } + h(x,t)$
$\alpha = \beta = 1$ and $\gamma = 0$	$\infty$-Laplacian: $-\Delta _{w,\infty } u(x) = h(x)$	$\partial _t u(x,t)=\Delta _{w,\infty } u(x,t) + h(x,t)$
$\alpha = \beta = 0$ and $\gamma = 1$	Laplace equation: $-\Delta _{w,2} u(x) = h(x)$	$\partial _t u(x,t)=\Delta _{w,2} u(x,t) + h(x,t)$
$\alpha = \beta = \frac{p-2}{p}$ and $\gamma = \frac{2}{p}$	Game *p*-Laplace equation: $-\Delta _{w,p}^{G} u(x) = h(x)$	$\partial _t u(x,t)=\Delta _{w,p}^{G} u(x,t) + h(x,t)$

For a complete presentation, let us say few words about the existence and uniqueness of a solution of equations of the form (10). In the elliptic case, existence of solutions can be proved using fixed point arguments and uniqueness relies on standard comparison results. While in the parabolic case, this can be done exploiting some properties shared by the averaging operators defined above. To this end, let us recall the following definition

#### **Definition 4**

(^21^) A continuous function $\mathcal {A}:\mathbb {R}^m\rightarrow \mathbb {R}$ is said to be an averaging operator if

- $$\mathcal {A}(0,\dotsc ,0) = 0$$ and $$\mathcal {A}(1,\dotsc ,1) = 1$$,
- $$\mathcal {A}(t x_1,\dotsc ,t x_m) = t\mathcal {A}(x_1,\dotsc ,x_m)$$ for all $$t\in \mathbb {R}$$,
- $$\mathcal {A}(t +x_1,\dotsc ,t+ x_m) = t+\mathcal {A}(x_1,\dotsc ,x_m)$$ for all $$t\in \mathbb {R}$$,
- $\mathcal {A}$ is nondecreasing with respect to each variable.

As an example, let us check these properties for the nonlocal mean operator. First, let us write $\text {NLM}(u)(x) = \mathcal {A}(u(y_1),\dotsc ,u(y_m))$ where we assume that $(y_i)_{i=1}^{m}$ are the neighbours of *x*. We clearly have $\mathcal {A}(0,\dotsc ,0) = 0$ and $\mathcal {A}(1,\dotsc ,1) = 1.$ Moreover, we have, for $t\in \mathbb {R}$

$$\begin{aligned} \mathcal {A}(tu(y_1),\dotsc ,tu(y_m)) = \frac{\sum _{j=1}^{m} w(x,y_j) (tu(y_j))}{\sum _{j=1}^{m} w(x,y_j)} = t \frac{\sum _{j=1}^{m} w(x,y_j) u(y_j)}{\sum _{j=1}^{m} w(x,y_j)} = t\mathcal {A}(u(y_1),\dotsc ,u(y_m)), \end{aligned}$$

and

$$\begin{aligned} \begin{aligned} \mathcal {A}(t+u(y_1),\dotsc ,t+u(y_m))&= \frac{\sum _{j=1}^{m} w(x,y_j) (t+u(y_j))}{\sum _{j=1}^{m} w(x,y_j)} = t \frac{\sum _{j=1}^{m} w(x,y_j) u(y_j)}{\sum _{j=1}^{m} w(x,y_j)}\\&= \frac{1}{\sum _{j=1}^{m} w(x,y_j)}\left( t \sum _{j=1}^{m} w(x,y_j) + \sum _{j=1}^{m} w(x,y_j) u(y_j)\right) \\&= t + \frac{\sum _{j=1}^{m} w(x,y_j) u(y_j)}{\sum _{j=1}^{m} w(x,y_j)} = t + \mathcal {A}(u(y_1),\dotsc ,u(y_m)). \end{aligned} \end{aligned}$$

Finally, since the weight function is positive, we deduce that $\mathcal {A}$ is nondecreasing. This shows that $\text {NLM}$ is as averaging operator in the sense of Definition 4. One can proceed similarly for $\text {NLD}$ and $\text {NLE}$, and as a consequence, $\text {NLA}$ is itself an averaging operator as a combination of averaging operators. To go further, let us assume for simplicity that $h\equiv 0$, then *u* solves

11 $$\begin{aligned} \partial _t u = \text {NLA}(u) - u~\text{ in }~\mathcal {V}~~\text{ and }~~u(x,0)= g~\text{ in }~\mathcal {V}, \end{aligned}$$

if and only if *u* solves the integral equation

$$\begin{aligned} \mathcal {K}_g u(x,t) = \int \nolimits _{0}^{t} e^{s-t}\mathcal {A}(u(y_1,s),\dotsc ,u(y_m,s))\textrm{d}s + e^{-t} u^{0}(x), \end{aligned}$$

where, again we denote $\text {NLA}(u)(x,s) = \mathcal {A}(u(y_1,s),\dotsc ,u(y_m,s))$ and $u^{0}:\mathcal {V}\rightarrow \mathbb {R}$ is a given function. Then, the existence and uniqueness of solutions to (11) can be obtained using a fixed point arguments on the operator $\mathcal {K}_g$ as done in^22^ for the case of trees.

To conclude this section, let us remark that just as their counterparts on Euclidean graphs, nonlocal PDEs on general weighted graphs can also be interpreted in terms of TOW games. To simplify the presentation, let us take $\alpha = \beta = 1$ and $\gamma = 0$, i.e., we are considering the equation $-\Delta _{w,\infty } u = h(x)$. Then, using Definitions 2–3, we easily see that this equation can be rewritten as^20^

12 $$\begin{aligned} u(x) = \max _{y\in \mathcal {V}}\left( \min _{z\in \mathcal {V}}\Big ( P(x,y,z)u(y) + (1-P(x,y,z))u(z)\Big )\right) , \end{aligned}$$

where $P(x,y,z) = \frac{\sqrt{w(x,y)}}{\sqrt{w(x,y)} + \sqrt{w(x,z)} }$. Now, think about the same configuration as the classical TOW game but with nonlocal neighbours, i.e., the token’s displacement takes place in $I(x_{k-1}) = \{x\in \mathcal {V}: w(x,x_{k-1})>0\}\cup \{x_{k-1}\}$ instead of $\epsilon$-balls with weight-dependent probabilities as follows. If the player ${\textrm{I}}$ wins the toss, the token will move to a position $x_{k}^{\textrm{I}}$ with probability $P(x_{k-1},x_{k}^{\textrm{I}},x_{k}^{\textrm{II}})$, and to a position $x_{k}^{\textrm{II}}$ with probability $1 - P(x_{k-1},x_{k}^{\textrm{I}},x_{k}^{\textrm{II}})$. This can be interpreted as a nonlocal TOW game, whose value function satisfies (12).

To summarize, so far we have established a unifying mathematical framework based on the TOW games (5) and (6), which allows one to recover a series of well-known PDEs on graphs, as depicted in Table 1. We have then extended this analogy to nonlocal PDEs on general weighted graphs, and shown that they could be implemented in a simple manner by resorting to nonlocal mathematical morphology operators. In the next section, we describe a few simple algorithms for inverse problems, which follow directly from this theoretical discussion.

## Simple algorithms for inverse problems

In this section we give simple algorithms based on the transcription of the TOW game on graphs, which will be used to solve a few inverse problems. As we shall see, the main features of these algorithms are their simplicity and ease of implementation, and the fact that many tasks can be achieved just by taking particular values of the TOW game parameters $\alpha ,\beta$ and $\gamma$.

### Unified interpolation for image and high dimensional data processing

We have seen that we are able to recover a bunch of local and nonlocal PDEs on graphs. We will use this as a methodology to solve several interpolation problems in image processing and machine learning. In particular, in the next section we will show examples in medical imaging and in cultural heritage corresponding to problems on graphs using PDEs of the form (8) to (9) with particular attention on colorization, inpainting and data classification. To do so we consider a subset $A\subset \mathcal {V}$ consisting of vertices with the missing information. To simplify the presentation suppose that $h\equiv 0$. Then, many problems we encounter in image processing and computer vision can be recast in the form of interpolation problems, i.e., one seeks constructing new values starting from known values, which amounts to solve the Dirichlet problem

13 $$\begin{aligned} \left\{ \begin{array}{ll} -\Delta _{\alpha ,\beta ,\gamma } u= 0&\quad\text{in}\quad A,\\ u= g&\quad\text{on}\quad \partial A, \end{array} \right. \end{aligned}$$

where $g:\partial A\rightarrow \mathbb {R}$ is the boundary data which depends on the considered application. To solve (13), the strategy is the following. We first consider the associated evolution problem:

14 $$\begin{aligned} \left\{ \begin{array}{ll} \partial _{t} u= \Delta _{\alpha ,\beta ,\gamma } u&\quad\text{in}\quad A,\\ u= g&\quad\text{on}\quad\partial A,\\ u_{\vert t=0}= u^0&\quad\text{in}\quad A, \end{array} \right. \end{aligned}$$

with some initial value $u^0: A\rightarrow \mathbb {R}$ chosen suitably for each application. As usual, (14) is solved using a Euler discretization by taking $\partial _{t}u \approx \frac{u_{n+1}-u_{n}}{\Delta t}$, where $u_{n}(x) = u(x,n\Delta t)$. Then (14) can be approximated via the following iterations:

$$\begin{aligned} \left\{ \begin{array}{ll} u_{0}= u^0&\quad\text{in}\quad A,\\ u_{n+1}= u_n + \Delta t \Delta _{\alpha ,\beta ,\gamma } u&\quad\text{in}\quad A,\\ u_{n+1}= g&\quad\text{on}\quad\partial A.\\ \end{array} \right. \end{aligned}$$

Taking $\Delta t = 1$ and $\Delta _{\alpha ,\beta ,\gamma } = \text {NLA}(u) - u$, we get a nonlocal average filter consisting of convex combination of the nonlocal, dilation, erosion and mean terms introduced in (10):

15 $$\begin{aligned} \left\{ \begin{aligned} u_{0}&= u^0&~\text{ in }~A.,\\ u_{n+1}&= \text {NLA}(u_n)&~\text{ in }~A,\\ u_{n+1}&= g&~\text{ on }~\partial A.\\ \end{aligned} \right. \end{aligned}$$

Thus, Problem (13) can be approximated via the iterative scheme (15) which consists of simple algebraic operations. This makes the approach convenient for users with no deep knowledge on PDEs to solve interpolation problems. Notice that this can also be applied to parabolic variants of (13).

### Unified segmentation and data clustering

For segmentation and data clustering, we make use of an adaptation of the eikonal equation on graphs. First, let us say that in this case one could make use of the iterative scheme (10) with $\alpha = \gamma = 0$ and $\beta = 1$ to solve the eikonal equation, however, for faster computations, it is preferable to use a monotone algorithm where we have an explicit formula for the solution^10^. Let $\mathcal {G}= (\mathcal {V},\mathcal {E},w)$ be a weighted graph and consider the following eikonal equation:

16 $$\begin{aligned} \left\{ \begin{aligned} \Vert (\nabla _{w}^{-}u)(x) \Vert _{\infty }&=h(x)&\text{ in }~\mathcal {V}\setminus S_0,\\ u&= 0&\text{ on }~ S_0, \end{aligned} \right. \end{aligned}$$

with $S_0 \subset \mathcal {V}$ the set of initial seed vertices. Using the operators defined in Definition 2, Eq. (16) becomes

17 $$\begin{aligned} \max _{y\in I(x)}\Big (\sqrt{w(x,y)}\max (0,u(x)-u(y) )\Big )=h(x). \end{aligned}$$

Then, setting $C = h(x),~k_i = \sqrt{1/w(x,y)}$, $a _i= \Big \{f(y_i):~y_i\in I(x_i)\Big \}$ and $n = \text {Card}(I(x))$, Eq. (17) becomes

$$\begin{aligned} \max _{i}\Big (\frac{(x-a_i)^{+}}{k_i}\Big )=C, \end{aligned}$$

whose unique solution is explicitly given by

$$\begin{aligned} x^* = \min _{i=1,\cdots ,n}(a_i + k_i C), \end{aligned}$$

which gives rise to a Dijkstra-like algorithm. Then, for semi-supervised graph clustering, this algorithm is extended by enabling label propagation on a general weighted graph (see^10^, Algorithm 2]). To recapitulate the process, we denote by $L = \{l_1,\cdots ,l_n\}$ the set of labels and by $S^{0} = \cup _{i=1}^{n}S_{i}^{0}$ the set of seeds, i.e., each $S_{i}^{0}$ is a set of vertices marked by a label $l_i$. Then, we mark each vertex $x\in \mathcal {V}$ with a label $l_i$ provided *x* is closer to a vertex of $S^{0}_{i}$ than other vertices of $S^0$ which decomposes the graph into *n* clusters. Each label $l_i$ is induced by a front $\mathcal {F}_{i}$ initialized at $\partial S_{i}^{0}$, and the final configuration $S_f$ is given by the set of vertices reached by $\mathcal {F}_i$ until it is stopped by the boundary of the domain or by another front. Note that this is a particular case ($p=\infty$) of the method to solve the eikonal equation with $\mathcal {L}_p$ norm^10^ . For $p=2$, it is worth mentioning that the obtained scheme on a *n*-dimensional grid in $R^n$ reduces to the celebrated Osher-Sethian scheme which can be solved using a Fast Marching method (FMM)^23^.

## Applications

In all the sequel, we are given a graph $\mathcal {G}= (\mathcal {V},\mathcal {E},w)$ constructed as described below. We will provide examples of nonlocal inpainting, colorization, semi-supervised segmentation using the iteration (15) as well as the Fast Marching-like algorithm described above.

The results of this section will mainly concern applications on cultural heritage and medical imaging. Cultural heritage applications will essntially focus on scenes taken from the Bayeux Tapestry. The Tapestry is a unique record of the Norman conquest of England and the battle of Hastings. It is an eleventh-century medieval embroidery of 70m long and 50cm tall, which has been digitized during a project gathering the City of Bayeux, the University of Caen-Normandy, the CNRS and the GREYC. The tapestry is of main interest to historians, archaeologists as well as textile researchers which makes all the methods and applications we propose useful since the tapestry in accessible to researchers only in a short period of the year. Concerning medical imaging, we shall focus on computer-aided cytology. Cytopathologists usually have to study the morphology and texture of cytoplasm and nuclei as imaged in microscopy to make a diagnosis decision. This task can be tedious since every slide supporting sample contains millions of cells and the presence of infected cells is rare. The use of PDEs on graphs for computer-aider cytology has been shown to be efficient for segmentation and semi-supervised classification of cells^24^. All the results concerning cytology are produced using the database of the Cherbourg University Hospital. A software called PdESigraph is available upon request and will be accessible online soon. It contains all the necessary material for non-mathematicians to construct the appropriate graphs and apply the presented algorithms for the different inverse problems.

### Construction of graphs

The first step is to construct an appropriate graph for the given data to apply the obtained algorithms. There are several well-known techniques to construct a weighted graph from a given discrete data set depending on whether the data is unorganized or structured. For unstructured data, we can mention $\epsilon$-neighbourhood graphs where we connect two elements $x,y\in \mathcal {V}$ by an edge if $\textrm{d}(x,y)\le \epsilon$ for some $\epsilon >0$, where $\textrm{d}$ is some distance on $\mathcal {G}$. We also find the k-nearest neighborhood graph (k-NNG) where each vertex is connected with its k nearest neighbours with respect to the distance $\textrm{d}$ (this will give rise to directed graphs due to the nonsymmetry of this neighborhood relationship, however undirected graph can still be obtained using a modification in the construction^25^).

Structured data (e.g., images, meshes), can naturally be described by grid graphs. In the case of images, each pixel is connected by an edge to adjacent vertices. Region adjacency graphs (RAG) were also designed for images where vertices correspond to image regions and edges are obtained by considering an adjacency distance. This can be generalized for other data, where a region $R_i$ is defined as a set of vertices such that $\mathcal {V}= \cup _{i} R_i$ and $\cap _{i} R_i = \emptyset$. Then, $R_i$ and $R_j$ are adjacent if there exists $x\in R_i$, $y\in R_j$ such that $x\sim y$ (see Fig. 1).

As for the weight function *w*, it is generally computed via a similarity map $s:\mathcal {E}\rightarrow \mathbb {R}^{+}$, so that $w(x,y) =s(x,y)$ if $(x,y)\in \mathcal {E}$ and 0 otherwise. In general we take

18 $$\begin{aligned} s(x,y) = 1,~s(x,y) = \exp \Big (\frac{-\textrm{d}(u^{0}(x),u^{0}(y))}{\sigma ^2}\Big ),~\text{ or }~s(x,y) = (\textrm{d}(u^{0}(x),u^{0}(y))+1)^{-1}. \end{aligned}$$

The choice depends on the application and can be, for example, either geometric (i.e., depending on the distance between pixels) or photometric (i.e., depending on the image or a characteristic vector). The role of similarity maps is to assign values close to 1 to similar vertices and values close to 0 to nonsimilar ones. For further derails and other constructions see e.g.,^26^. Let us stress that the choice of the parameter $\sigma$ in (18) can be done globally or locally, i.e., by assigning to each vertex a parameter $\sigma _x$ depending on the application. For images, we generally make use of patches as follows. Pick some vertex $x\in \mathcal {V}$ and consider a square *C*(*x*, *n*) of size $n^2$ centred at *x*. Then $\mathcal {P}(x) = \left( u^{0}(y)\right) ^{T}_{y\in C(x,n)}$ is called a patch centred at *x*. Using this definition, one can define a similarity map via $s(x,y) = \exp \Big (\frac{-\Vert \mathcal {P}(x)-\mathcal {P}(y)\Vert _{2}^{2}}{\sigma ^2}\Big )$. This notion can be extended to the case of 3-dimensional points clouds^27^.

### Nonlocal inpainting

Inpainting is a virtual restoration processes. It consists in reconstructing a damaged or incomplete part of an image. Most of the proposed algorithms to perform this task are based on PDEs and variational formulations^28,29^. Nonlocal approaches for image inpainting have been widely considered since the work on nonlocal filtering^30^. This approach was particularly successful for the treatment of textured images, for which local methods had many limitations. To formulate the problem within our framework, we consider $A\subset \mathcal {V}$ the set of vertices with missing data and prescribe boundary condition on $\partial A$ via a function $g:\partial A\rightarrow \mathbb {R}^{c}$, with *c* the number of color channels of the image ($c = 3$ in the examples below). We apply the iterative scheme (15) with $\alpha = \beta = 1, \gamma = 0$, i.e., the $\infty$-Laplacian is used for the inpainting of 2D images and 3D point clouds from scribbles giving reference colors.

**Figure 2:**
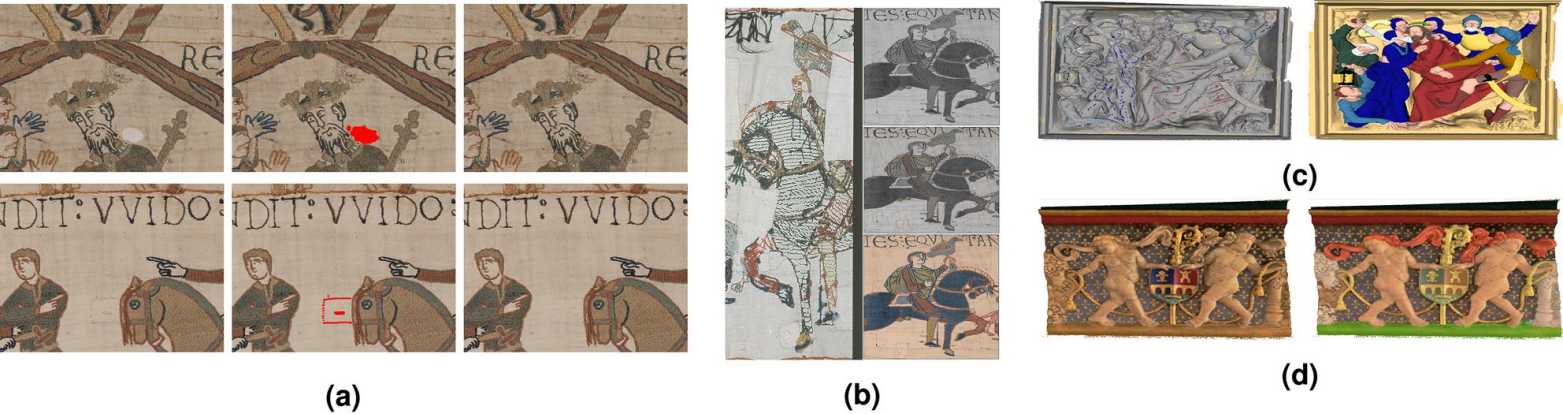
Virtual restoration and colorization. (**a**) 2D restoration (original images, images with areas to be restored indicated in red, and restoration results). (**b**) 2D colorization, where the left column represents the backside of a scene from the Bayeux Tapestry, and the second column represents the initial image, the image with seeds, and the colored result. (**c**, **d**) 3D colorization, with input models on the left and colored ones on the right.

In Fig. 2a we use our approach for the virtual restoration of a 2D image of King Edward taken from the Bayeux Tapestry. A part of this scene was damaged due to wax stains caused by candles when the Tapestry was at the cathedral. As one sees, the proposed algorithm allows “virtually restoring” the part which has been damaged.

### 2D-3D colorization

Colorization consists in adding colors to monochromatic or colorless images. It can be particularly helpful in cultural heritage and movie industry applications, or in conjunction with 3D acquisition techniques. Colorization of monochromatic images is based on the intensity channel to determine similarities between pixels and proceed to color diffusion from scribbles. For 3D data, the intensity channel is missing and one needs to proceed differently. An optimization-based approach was proposed for 3D meshes colorization^31^ relying on similarities between mesh vertices via the spin image descriptor^32^. Yet, this approach seems not suitable for 3D point clouds as they have no intrinsic connectivity. On the other hand, formulating 3D colorization as an interpolation problem allows us exploiting the simple nonlocal averaging iterations (15), starting from a few user-specified color scribbles.

For 2D images, we apply our schemes to recover the original colors (at least vivid ones) of the Bayeux Tapestry. This is done using a ”reflection” trick as follows. In fact, the colors in the exposed side of the Tapestry tend to bleed and fade over time due to sunlight, humidity and reaction with chemicals such as oxygen, ozone etc. Colors such as blue, red, orange, dark green and brown are more susceptible to this. Since the backside of the Tapestry is less exposed, one can guess the original colors from the yarns in the back of the embroidery. We exploit this to use appropriate seeds for the colorization process. This is illustrated in the right column of Fig. 2b where we find the initial image, image with seeds and the colored images where the seeds are take from the left column of Fig. 2b. The top row in Fig. 2c illustrates virtual colorization of a 3D model representing the ”Betrayal of Judas”^33^ using the proposed scheme (more particularly, the $\infty$-Laplacian). A detailed comparison between the obtained results (with different values of $\alpha ,\beta$ and $\gamma$) and an alternative approach^31^ can be found in a dedicated paper^20^. The bottom row in Fig. 2d illustrates the colorization of a 3D model from the chimney of the old Abbaye du voeu at Cherbourg^34^ and in particular, we see that the method can be applied to change colors.

### Semi-supervised segmentation and data clustering

Image segmentation consists in partitioning images into multiple regions to make them easier to analyse. In particular, one wishes to detect objects such as roads in satellite images, tumors in medical imaging, etc. As for data clustering, it consists in splitting data into different families of objects sharing similar properties, such as infected cells in a medical database, etc. All these tasks can be achieved using the eikonal Eq. (16), with $h\equiv 1$ and labels propagation as described before. One of the advantages of this formulation is that it allows segmentation on any graph representing images. In the examples below, we use a 4-adjacency grid graph to build an image partition. The obtained region map is then turned into a RAG. To allow labels growing beyond local neighbors, each vertex neighbor is extended by a *k*-NN based on mean color value.

**Figure 3:**
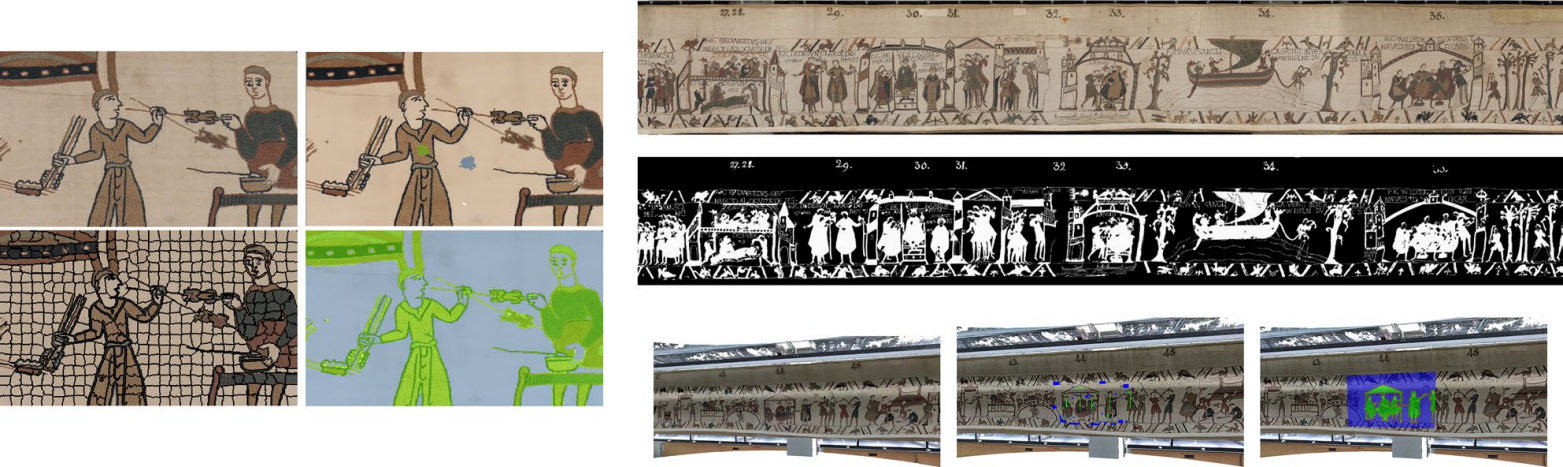
Semi-supervised segmentation of the Bayeux Tapestry. Left: superpixel-based segmentation of a single scene (initial image, image with seeds, image partition with supervertices using a regular grid of seeds, and segmentation result). Right, first and second rows: segmentation of the foreground on 2D images (initial image and segmentation result). Right, third row: 3D point cloud segmentation (from left to right: initial image, image with initial labels, segmentation result).

In the following examples, we perform segmentation using superpixels decomposition which allows reducing image complexity by regrouping pixels in a region map while preserving contours. This can be done by dilating a regular grid of seeds by the label propagation method using the eikonal equation^10^ in such a way to preserve the local structure of the image. This is illustrated in Fig. 3, left, where we show the segmentation of 2D and 3D scenes from the Bayeux Tapestry.

**Figure 4:**
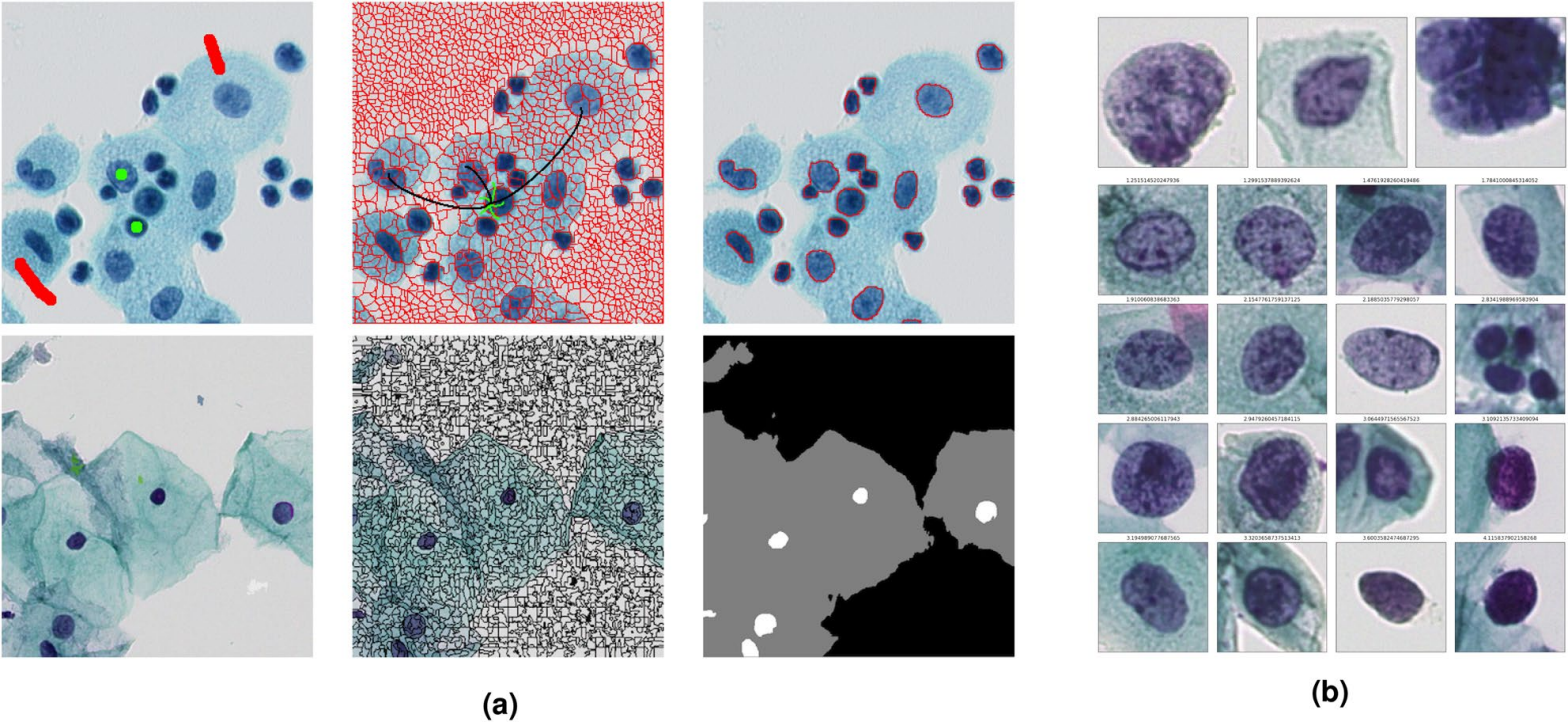
Applications in medical imaging. (**a**) Semi-supervised segmentation of cell images (from left to right: images with initial labels, superpixel segmentation and segmentation results. (**b**) Indexation using the eikonal equation (top: abnormal cells used as seeds; bottom: some indexed cells from the database).

Figure 4a shows how our framework can be used for segmenting medical images. In fact, each image can contain various objects to be segmented. In particular, nuclei are numerous and not adjacent, but some are still concentrated in some regions and are separated by the cytoplasm or the background. This suggests considering nuclei as a first class and the background and cytoplasm as the second class. The nonlocal graph structure is needed to allow labels grow beyond local neighbours. To this end, the eikonal equation is used twice: first, for the superpixel decomposition, and then for segmentation. Thanks to this approach, only few seeds are needed to perform efficient segmentation of images containing multiple and non-adjacent objects to detect. Another application we can consider is indexation. In fact, after segmenting cells using the eikonal equation, we get a database of cells which can be represented in the form of a graph. The top of Fig. 4b illustrates some abnormal cells that will be used as seeds. Then, using the eikonal equation one can compute the distance map and thus, find the closest cells to the abnormal cells, as illustrated in the bottom of Fig. 4b where we can see 16 cells amongst the database that are the closest to the seeds.

**Figure 5:**
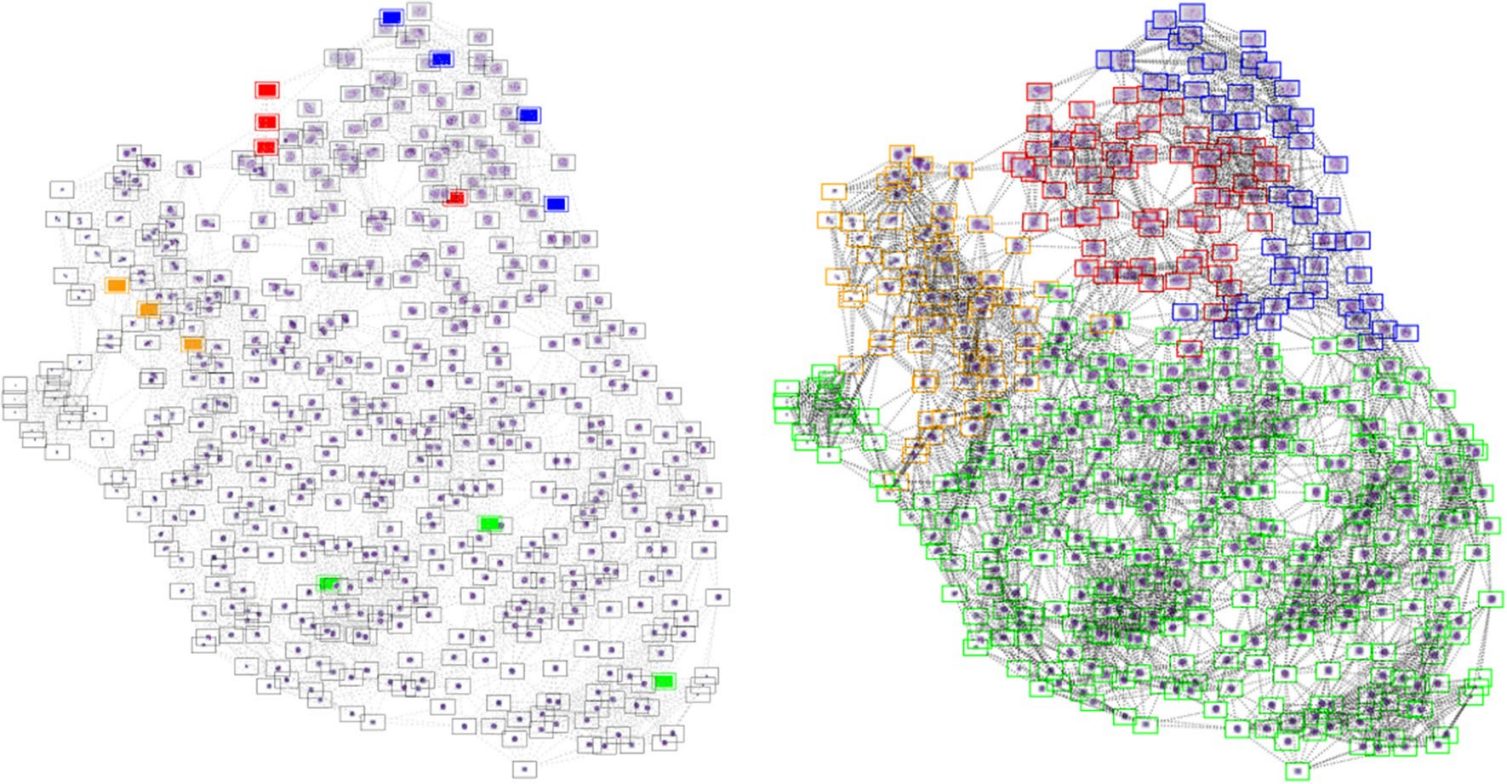
Illustration of semi-supervised data clustering using the eikonal equation. Left: graph built on a database of cytological cells, where some cells are initially labelled (blue, green, red). Right: final clustering.

We conclude this section by applying our approach to semi-supervised classification of cells in a cytological slide into normal and abnormal classes. We first perform segmentation using the eikonal equation as discussed above to extract nuclei. We used $10\%$ seeds on a dataset of 3956 cells extracted from a cytological slide divided by the cytologists into 4 classes where nuclei are described by characteristics concerning the form, surface, color, and the texture of cells, etc. Then we perform label propagation using the eikonal equation (with $p=1,2$) on a nearest neighbour graph as described in the previous section. This gives the result illustrated in Fig. 5 which are promising since we get $98.47\%$ classification accuracy for $p=1$ and $98.27\%$ for $p=2$. These algorithms are present in the Antarctic software, which was developed during the phd thesis of X. Desquenes^35^ to provide an accessible support to pathologists for the classification and the segmentation of cytological slides. The software comes with a database of millions of cells and allows pathologists to create and modify characteristics.

## Discussion

In this note, we revisited the notion of tug-of-war games and related PDEs on graphs. We showed that the transcription of such games on graphs allows recovering a bunch of nonlocal elliptic and parabolic PDEs on graphs. We used this observation as a unified methodology to solve interpolation problems. This gives rises to simple algorithms for image and high dimensional data processing. We illustrated this approach by examples from cultural heritage and medical imaging. In future work we are planing to consider other real life applications and compare results with the related works. In addition, the continuum limit of the considered PDEs, as the number of vertices goes to infinity, will be investigated in depth.

## Data Availability

The datasets used and/or analysed during the current study available from the corresponding author on reasonable request.
